# Soldier’s Friend

**Published:** 1861-07

**Authors:** 


SOLDIER'S FRIEND.—Under this title Dr. Ziegler, of this city, has written a small tract, of a seasonable character, published by S. S. White, for gratuitous distribution among soldiers. The hints he has thrown out are of a highly practical character, comprehensive and easily understood. They are well adapted for the purposes designed, and will no doubt prove very useful to those who are in the field. The tract is worthy of extended circulation.

